# S11. BRAIN ABNORMALITIES ASSOCIATED WITH DISEASE-SPECIFIC AND GENETIC RISK FACTORS FOR SCHIZOPHRENIA

**DOI:** 10.1093/schbul/sby018.798

**Published:** 2018-04-01

**Authors:** Vina Goghari

**Affiliations:** University of Toronto

## Abstract

**Background:**

Schizophrenia is a severe disorder affecting approximately 1% of the population. The disorder is associated with symptoms such as false perceptions and beliefs and disturbances in affect and language production. In 2004 the total direct healthcare and non-healthcare cost in Canada was estimated at $2 billion, with an additional productivity loss estimated at $5 billion.

**Methods:**

I will present a program of research into disease-specific and genetic risk factors associated with structural and functional brain abnormalities, including morphology (amount of grey matter), structural connectivity (amount of white matter integrity), and brain functioning (amount of brain activity) in individuals with schizophrenia, their family members, and community controls using magnetic resonance imaging. As healthy relatives share genes with their affected family member, but do not share the disease process, abnormalities present in relatives are likely associated with the genes for schizophrenia.

**Results:**

Evidence was found for disease-specific, genetic risk and compensatory brain mechanisms associated with schizophrenia that were complementary between the results from brain morphology, structural connectivity, and brain functioning.

**Discussion:**

Isolating the biological and genetic basis of these deficits could ultimately aid in developing novel psychosocial and pharmacological treatments to facilitate improved day-to-day functioning in schizophrenia.

